# By modulating miR‐525‐5p/Bax axis, LINC00659 promotes vascular endothelial cell apoptosis

**DOI:** 10.1002/iid3.764

**Published:** 2023-01-18

**Authors:** Xizheng Zhu, Beijia Chen, Hui Xu

**Affiliations:** ^1^ Department of Interventional Radiology Wuhan Asia General Hospital Wuhan China; ^2^ Department of Cardiology Fifth Hospital in Wuhan Wuhan China

**Keywords:** deep vein thrombosis, LINC00659, miR‐525‐5p/Bax axis, vascular endothelial cell apoptosis

## Abstract

**Background:**

Deep vein thrombosis (DVT) is a vascular disease that has no effective treatment at present. Endothelial cells play a crucial role in the processes vasoconstriction, platelet activation, and blood coagulation and are an integral part of the vascular response to injury resulting in thrombus formation.

**Objective:**

The aim of this study was to investigate the roles and mechanisms of long noncoding RNA LINC00659 (LINC00659) in endothelial cells.

**Methods:**

The functions of LINC00659 and miR‐525‐5p on endothelial cells were explored by cell transfection assays, and the expression levels of LINC00659, miR‐525‐5p, and Bax in human umbilical vein endothelial cells (HUVECs) were assessed with reverse transcriptase‐quantitative polymerase chain reaction (RT‐qPCR). Binding sites of LINC00659 and miR‐525‐5p were subsequently analyzed with bioinformatics software, and validated with dual‐luciferase reporter gene assay. Effects of LINC00659 and miR‐525‐5p on proliferation and apoptosis of HUVECs were detected with MTT (3‐(45)‐dimethylthiahiazo (‐z‐y1)‐35‐di‐phenytetrazoliumromide) assay and flow cytometry. RT‐qPCR and western blot analysis were used to evaluate the mRNA and protein levels of apoptosis‐related markers Bcl‐2 and Bax in HUVECs.

**Results:**

LINC00659 directly targeted and negatively regulated miR‐525‐5p, and Bax was a target of miR‐525‐5p. Upregulation of LINC00659 could inhibit proliferation and promote apoptosis of HUVECs, while the silencing of LINC00659 could increase the viability of HUVECs and inhibit apoptosis via upregulating miR‐525‐5p. Further mechanistic studies revealed miR‐525‐5p could negatively regulate Bax in HUVECs, and increased the viability of HUVECs and inhibited apoptosis by downregulating Bax expression.

**Conclusion:**

LINC00659 played an important role in DVT by regulating the apoptosis of vascular endothelial cells through regulating miR‐525‐5p/Bax axis.

## Graphical abstract

Our study showed that silencing of LINC00659 could play important roles in deep vein thrombosis (DVT) through regulating miR‐525‐5p/Bax axis to inhibit vascular endothelial cell apoptosis. This finding provides the new potential target and direction for the treatment and diagnosis of DVT.

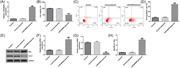

## 1 INTRODUCTION

Deep vein thrombosis (DVT) is a blood clot that forms in a vein, and is also one of vascular diseases in the world.^1^ DVT often occurs in the lower extremities, with clinical manifestations of lower extremity swelling, superficial varicose veins, pigmentation, and chronic pain, which have significantly affected the quality of patient's life and led to death.^2^ Currently, effective treatments for DVT include surgical treatment and nonsurgical treatment, among which drug therapy is a common nonsurgical treatment method, mainly including thrombolytic therapy, anticoagulant therapy, antiplatelet drugs, and vasodilator drugs.^3^ However, these treatments have low cure rates and suffer from various side effects. Therefore, exploring the underlying molecular mechanisms and potential therapeutic approaches of DVT is necessary.

The main causes of DVT include venous wall damage, slow blood flow, and hypercoagulability.^4^, ^5^ Among them, vascular wall damage is an important cause of venous thrombosis.^6^ A large number of research have confirmed that vascular endothelial cells are closely related to the formation of DVT.^7^ Endothelial cells cover the surface of all blood vessels.^8^ They provide an important barrier between the cellular and noncellular components of circulating blood and the mesenchyme; regulate tissue perfusion and provide oxygen and nutrition; together with underlying smooth muscle cells and pericellular endothelial cells, they help to recruit inflammatory cells and control blood pressure.^9^ The basic role of endothelial dysfunction in cardiovascular diseases (including hypertension, coronary artery disease, and peripheral artery disease) has been confirmed in many clinical and experimental studies.^10^, ^11^ Endothelial cells play a crucial role in all these processes, including vasoconstriction, platelet activation, and blood coagulation, and are part of the vascular damage response leading to thrombosis.^12^ Apoptosis of vascular endothelial cells greatly decreases their ability to release active substances, destroy the stability of the anticoagulant and fibrinolytic system, and weakens their multiple defences in the vasculature, ultimately leading to the formation of thrombus.^13^, ^14^ Endothelial cells are increasingly considered as potential targets to prevent thrombotic events and accelerate thrombolysis.

Long noncoding RNAs (lncRNAs) are a class of noncoding RNA molecules with a length of more than 200 nt do not encode proteins, and can regulate gene expression.^15^, ^16^, ^17^ So far, a variety of lncRNAs have been reported to be aberrantly expressed in vascular diseases, and may be involved in regulating vascular disease development.^16^, ^18^, ^19^, ^20^ For example, lncRNA MALAT1 was recently found to inhibit the proliferation of endothelial progenitor cells (EPCs) in DVT through regulating Wnt/β‐catenin signaling pathways.^21^ A recent study has analyzed the underlying mechanisms of lncRNAs and miRNAs in venous thrombosis through transcriptome profiling, and identified lncRNAs as biomarkers for DVT.^22^ LINC00659, a novel lncRNA involved in tumorigenesis, acts as the oncogene to regulate cell growth in colorectal cancer.^23^, ^24^ Recent findings have shown that LINC00659 is significantly overexpressed in patients with DVT.^22^ However, the potential function of LINC00659 in DVT is unclear.

MicroRNAs (miRNAs) are the class of evolutionarily conserved noncoding RNAs with the function of regulating gene expression at the translational level.^25^, ^26^ MiRNAs were reported to play important roles in development of DVT, and lncRNAs were involved in regulation of DVT as competitive RNAs (ceRNAs) for miRNAs.^13^, ^22^, ^27^, ^28^, ^29^ Study has shown that GUSBP5‐AS regulates angiogenesis and proliferation of EPCs through miR‐223‐3p, and alleviates development of DVT.^30^ In addition, downregulation of LINC01123 inhibited DVT formation via miRNA‐125a‐3p.^28^ In this study, we found a potential binding site between LINC00659 and miR‐525‐5p through bioinformatics software analysis. However, whether LINC00659 is involved in DVT development by regulating miR‐525‐5p is not clear.

Human umbilical vein endothelial cell (HUVEC) is widely used to establish an in vitro experimental model for exploring vascular endothelial cells.^31^ The apoptosis of HUVEC has been proved to be a mechanism of many cardiovascular diseases (including thrombosis), which can cause endothelial dysfunction and other complications related to vascular diseases.^32^, ^33^ At present, HUVEC is widely used to study DVT in vitro.

This study aimed to investigate whether LINC00659 has a certain effect on vascular endothelial cells, and to analyze the underlying molecular mechanisms, providing a new potential target for the treatment of DVT.

## 2 MATERIALS AND METHODS

### 2.1 Cell culture

HUVECs were purchased from American Type Culture Collection. HUVECs were cultured with Dulbecco's modified Eagle medium (DMEM; Basal Media) medium containing 10% fetal bovine serum (FBS; Biological Industries). Cells were placed in a 5% CO_2_, 37°C incubator, and changed medium every other day.

### 2.2 Experimental design and cell transfection

Control‐siRNA, LINC00659‐siRNA, inhibitor control, miR‐525‐5p inhibitor, control‐plasmid, LINC00659‐plasmid, mimic control, and miR‐525‐5p mimic were obtained from Ribobio. To explore the effects of LINC00659 and miR‐525‐5p on HUVECs, HUVECs were transfected with control‐plasmid, LINC00659‐plasmid, control‐siRNA, LINC00659‐siRNA, inhibitor control, miR‐525‐5p inhibitor, LINC00659‐siRNA + inhibitor control, LINC00659‐siRNA + miR‐525‐5p inhibitor group, mimic control, miR‐525‐5p mimic, miR‐525‐5p mimic + control‐plasmid, or miR‐525‐5p mimic + Bax‐plasmid with Lipofectamine™ 2000 (Invitrogen) according to the manufacturer's instructions. After 48 h of transfection, the transfection efficiency was measured through reverse transcriptase‐quantitative polymerase chain reaction (RT‐qPCR).

### 2.3 RNA extraction and RT‐qPCR

Total RNAs were isolated from HUVECs with TRIzol kit (Vazyme) according to the manufacturer's instructions. Extracted total RNAs were reverse transcribed into cDNA with PrimeScript® RT Master Mix Kit (Takara). Subsequently, qPCR was performed with SYBR® Premix Ex TaqTM II (Takara) following the manufacturer's instructions, and the relative mRNA expression of LINC00659, miR‐525‐5p, Bax, and Bcl‐2 were calculated with $2^{‐∆∆C_{t}}$ method. U6 and glyceraldehyde‐3‐phosphate dehydrogenase (GAPDH) were used as the internal controls. The primer sequences were as following: LINC00659 forward, 5′‐ ACCCCTGAAGGACCATATCCA‐3′, LINC00659 reverse, 5′‐ GGCTCGGCTGTGTCTCAAG −3′; miR‐525‐5p forward, 5′‐ GTCGTATCCAGTGCGTGTCGTG −3′, miR‐525‐5p reverse Forward, 5′‐ GCGAGCACAGAATTAATACGACT ‐3′; Bax Forward, 5′‑TGG CAG CTG ACA TGT TTT CTGAC‑3′, Bax Reverse, 5′‑TCA CCC AAC CAC CCT GGT CTT‑3′; Bcl‐2 Forward Forward, 5′‑ GCC CTG TGG ATG ACTGAGTA‑3′, Bcl‐2x Reverse, 5′‑ GGC CGT ACA GTT CCA CAA AG‑3′; U6 Forward, 5′‐CTC GCT TCG GCA GCA CA‐3′, U6 reverse, 5′‐AAC GCT TCA CGA ATT TGC GT‐3′; GAPDH forward, 5′‐ACACCCACTCCTCCACCTTT‐3′, GAPDH reverse, 5′‐TTACTCCTTGGAGGCCATGT‐3′.

### 2.4 MTT assay for cell proliferation

Cell proliferation was detected with MTT assay according to previous study.^34^ Briefly, cells were seeded into 96‐well plates at 2000 cells/well. Then, 10 μl MTT solution (5 μg/μl; Beyotime) was added to each well and incubated for 4 h in a 37°C, 5% CO_2_ incubator. Subsequently, 100 μl Dimethyl sulfoxide (DMSO) was added to each well, mixed and incubated in an incubator until Formazan was completely dissolved. The optical density  values were determined at a wavelength of 570 nm with a Fluoroskan FL microplate reader (Thermo Scientific).

### 2.5 Apoptosis detection with flow cytometry

Apoptosis was detected with the Annexin V‐fluorescein isothiocyanate/propidium iodide (PI) Apoptosis Detection Kit (Beyotime) according to the manufacturer's instructions. Briefly, cells were digested with trypsin and collected by centrifugation at 300*g* for 5 min. After washing twice with phosphate buffer saline, 100 µl binding buffer was added to resuspend the cells. Then, the cells were incubrated with 5 μl Annexin V and 10 µl PI for 15 min at room temperature in the dark. Finally, the cells were detected using a flow cytometer (BD Biosciences).

### 2.6 Western blot assay

Total proteins were extracted from HUVECs with radioimmune precipitation assay buffer (Solarbio), and the amount of protein was measured with a BCA kit (Thermo Fisher Scientific) according to the manufacturer's instructions. Equal amounts of protein were separated via 12% sodium dodecyl sulfate‐polyacrylamide gel electrophoresis, and then transferred onto polyvinylidene fluoride membranes (Millipore). The membranes were then blocked with 5% milk for 1 h and incubated overnight at 4°C with primary antibodies, including anti‐rabbit‐Bcl‐2 (ab59348; Abcam), anti‐rabbit Bax (2772S; Cell Signaling Technology), and anti‐rabbit GAPDH (5174; Cell Signaling Technology). The next day, the membranes were washed three times with TBST buffer and then incubated with horseradish peroxidase‐labeled secondary antibodies for 2 h. Finally, protein bands were visualized with electrochemiluminescence (ECL) luminescent solution.

### 2.7 Dual‐luciferase reporter gene assay

The starBase v2.0 database (http://starbase.sysu.edu.cn/) and the TargetScan website (www.targetscan.org) were used to predict binding sites between LINC00659 and miR‐525‐5p, and miR‐525‐5p, and Bax. Subsequently, dual luciferase reporter assays were performed as previously described.^35^ Briefly, 293T cells were cotransfected with LINC00659‐WT (Bax‐WT) or LINC00659‐MUT (Bax‐MUT), and miR‐525‐5p mimic or mimic control with Lipofectamine 2000 (Invitrogen). After 48 h of transfection, luciferase activities were analyzed with dual luciferase reporter assay system (Promega) as per the manufacturer's instructions.

### 2.8 Statistical analysis

All data were analyzed with SPSS 19.0 statistical software (IBM Corp.) and expressed as the mean ± standard deviation (SD). The differences between groups were tested with one‐way aalysis of variance followed by Tukey's test or the Student's *t* test. The *p* < .05 was considered a statistically significant difference.

## 3 RESULTS

### 3.1 Effects of LINC00659 on the viability and apoptosis of HUVECs

**Figure 1:**
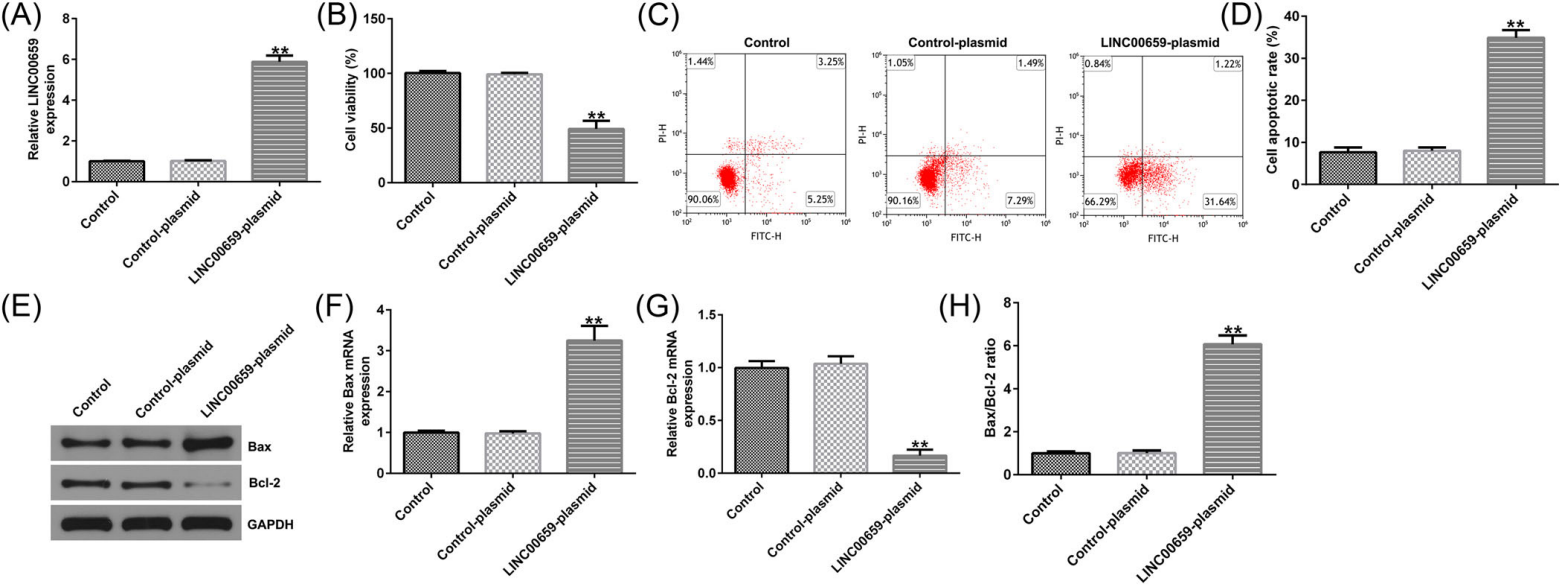
Effects of LINC00659 on the proliferation and apoptosis of HUVECs. (A) The expression of LINC00659 was detected by RT‐qPCR; (B) the cell proliferation of HUVECs was assessed by MTT; (C, D) the apoptosis of HUVECs was detected by flow cytometry; (E–G) the protein and mRNA levels of Bax and Bcl‐2 were analyzed by western blot analysis and RT‐qPCR. (H) Bax/Bcl‐2 ratio was presented. **p < .01 versus control‐plasmid group. HUVEC; human umbilical vein endothelial cell; RT‐qPCR, reverse transcriptase‐quantitative polymerase chain reaction.

To explore roles of LINC00659 in HUVECs, we transfected control‐plasmid or LINC00659‐plasmid into HUVECs. The results showed that LINC00659‐plasmid could effectively enhance the level of LINC00659 in HUVECs (Figure 1A). In addition, results of MTT assays showed that LINC00659 overexpression could significantly inhibit cell viability of HUVECs (Figure 1B). Furthermore, we detected apoptosis through flow cytometry, and the results showed that upregulation of LINC00659 remarkably induced apoptosis of HUVECs (Figure 1C,D). Moreover, overexpression of LINC00659 significantly increased Bax (Figure 1E,F), decreased Bcl‐2 (Figure 1E,G), and increased Bax/Bcl‐2 ratio (Figure 1H) in HUVECs. These results suggest that LINC00659 could inhibit cell viability and promoted apoptosis in HUVECs.

### 3.2 MiR‐525‐5p directly targets LINC00659

**Figure 2:**
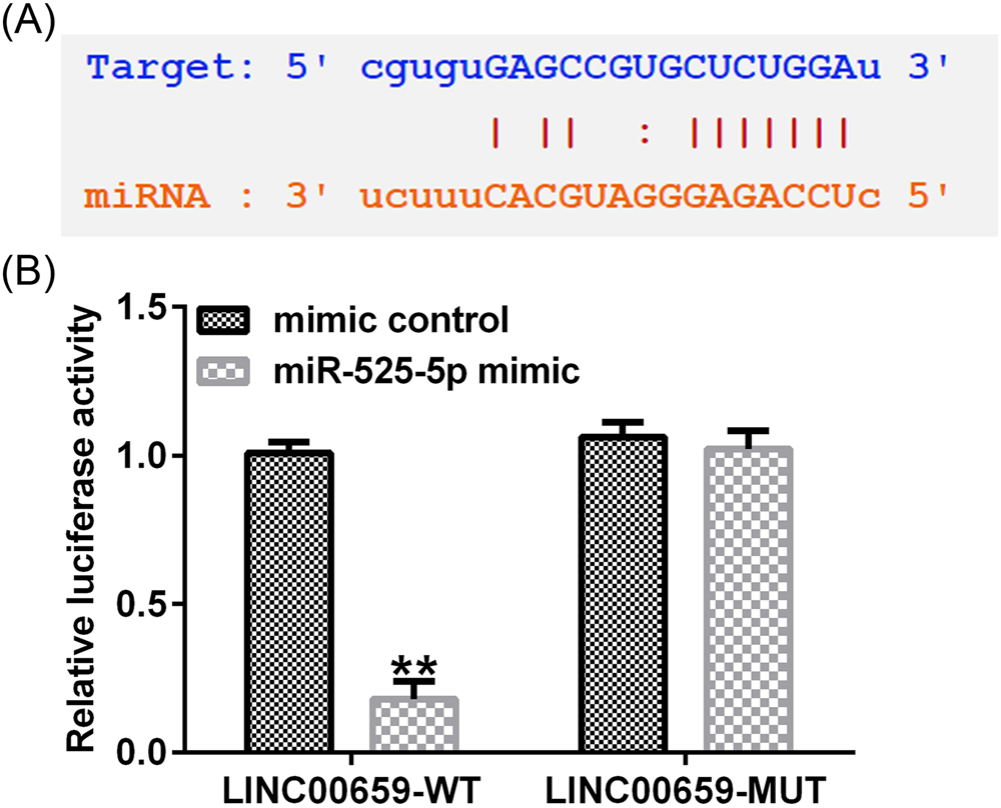
Determination of the relationship between LINC00659 and miR‐525‐5p. (A) StarBase predicts the binding sites between LINC00659 and miR‐525‐5p; (B) the interaction between LINC00659 and miR‐525‐5p is verified through dual‐luciferase reporter assay. **p < .01 versus mimic control.

Starbase database showed the binding sites between LINC00659 and miR‐525‐5p (Figure 2A). Subsequently, relationship between LINC00659 and miR‐525‐5p was verified via dual‐luciferase reporter assay. Compared to the mimic control group, miR‐525‐5p mimic could notably reduce the luciferase activity of LINC00659‐WT, whereas the luciferase activity of LINC00659‐MUT had no significant change (Figure 2B). These results indicated miR‐525‐5p is a direct target of LINC00659.

### 3.3 LINC00659 negatively regulates miR‐525‐5p expression in HUVECs

**Figure 3:**
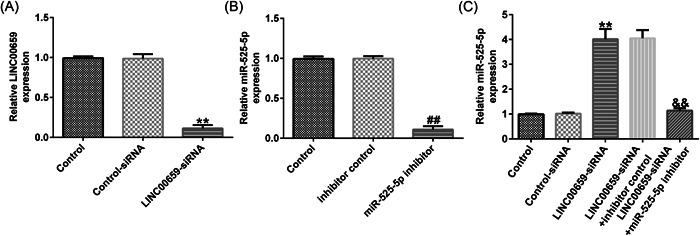
LINC00659 negatively regulates miR‐525‐5p expression in HUVECs. (A–C) The expression of LINC00659 and miR‐525‐5p were detected by RT‐qPCR. **p < .01 versus control‐siRNA; ^##^ *p* < .01 versus inhibitor control; ^&&^ *p* < .01 versus LINC00659‐siRNA + inhibitor control. HUVEC; human umbilical vein endothelial cell; RT‐qPCR, reverse transcriptase‐quantitative polymerase chain reaction.

To further investigate the relationship of LINC00659 and miR‐525‐5p in HUVECs, we inhibited the expression of LINC00659 by transfecting LINC00659‐siRNA into HUVECs. Additionally, LINC00659‐siRNA was cotransfected with miR‐525‐5p inhibitor or control inhibitor into HUVECs. The results demonstrated compared with the control‐siRNA group, LINC00659‐siRNA significantly reduced the level of LINC00659 in HUVECs (Figure 3A), and miR‐525‐5p inhibitor significantly reduced expression of miR‐525‐5p in HUVECs (Figure 3B). Silencing of LINC00659 notably enhanced the expression of miR‐525‐5p in HUVECs, which was remarkably abolished by miR‐525‐5p inhibitor cotransfection (Figure 3A).

### 3.4 Silencing of LINC00659 increases viability and inhibits apoptosis by upregulating miR‐525‐5p expression in HUVECs

**Figure 4:**
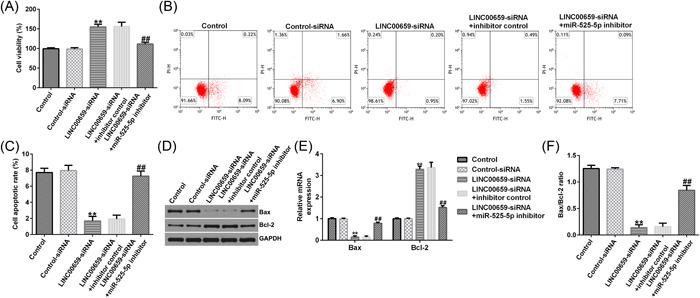
LINC00659 silencing affects the proliferation and apoptosis of HUVECs by upregulating miR‐525‐5p. (A) MTT assay was used to evaluate the cell proliferation of HUVECs; (B, C) flow cytometry was used to detect the apoptosis of HUVECs; (D and E) the protein and mRNA levels of Bax and Bcl‐2 were detected by western blot analysis and RT‐qPCR. F. Bax/Bcl‐2 ratio was presented. **p < .01 versus control‐siRNA; ^##^ *p* < .01 versus LINC00659‐siRNA + inhibitor control. HUVEC; human umbilical vein endothelial cell; RT‐qPCR, reverse transcriptase‐quantitative polymerase chain reaction.

Subsequently, we evaluated functions of LINC00659 and miR‐525‐5p on cell proliferation and apoptosis of HUVECs. Results indicated that downregulation of LINC00659 could enhance cell viability and reduce apoptosis of HUVECs, and these effects were significantly abolished by miR‐525‐5p inhibitor (Figure 4A–C). Furthermore, results of RT‐qPCR and western blot demonstrated downregulation of LINC00659 reduced Bax (Figure 4D,E), increased Bcl‐2 expression (Figure 4D,E), and decreased Bax/Bcl‐2 ratio (Figure 4F) in HUVECs. While all these changes were reversed by miR‐525‐5p inhibitor. These results suggest that downregulation of LINC00659 can enhance the viability of HUVECs and inhibit apoptosis by upregulating miR‐525‐5p.

### 3.5 Bax is the direct target of miR‐525‐5p

**Figure 5:**
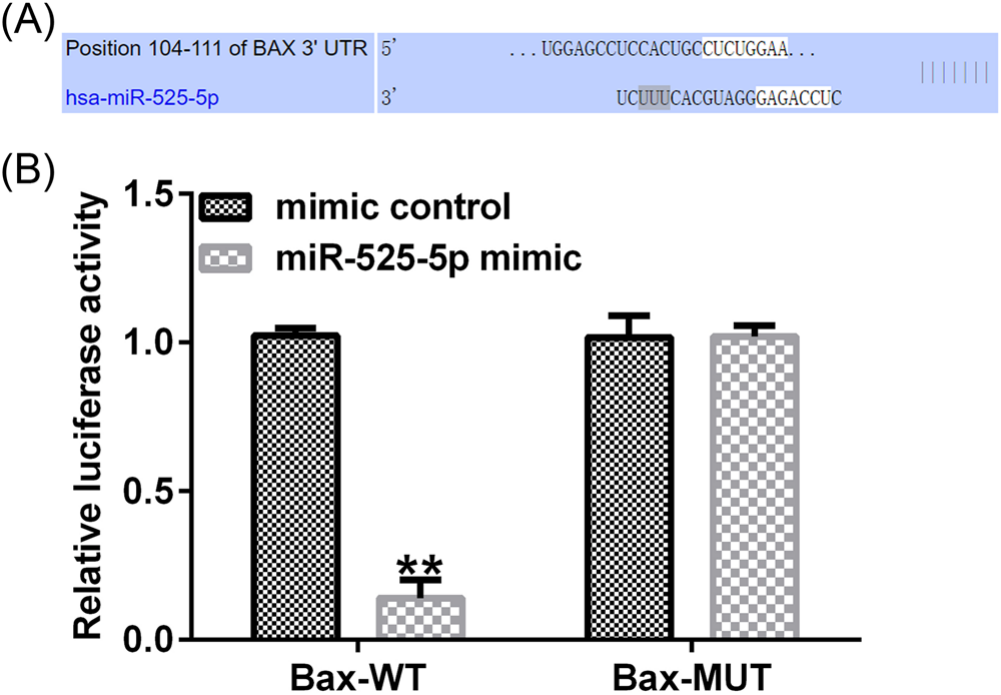
(A) Bax is a direct target of miR‐525‐5p. TargetScan database predicted binding sites between miR‐525‐5p and Bax; (B) the interaction between miR‐525‐5p and Bax was verified with dual‐luciferase reporter assay. **p < .01 versus mimic control.

Next, we analyzed potential target genes of miR‐525‐5p by bioinformatics software. TargetScan database predictions showed the binding sites between miR‐525‐5p and Bax (Figure 5A). Dual‐luciferase reporter assay showed that miR‐525‐5p mimic effectively inhibited luciferase activity of Bax‐3′UTR‐WT in 293 T cells (Figure 5B). These results suggest that Bax is a direct target of miR‐525‐5p.

### 3.6 MiR‐525‐5p negatively regulates Bax expression in HUVECs

**Figure 6:**
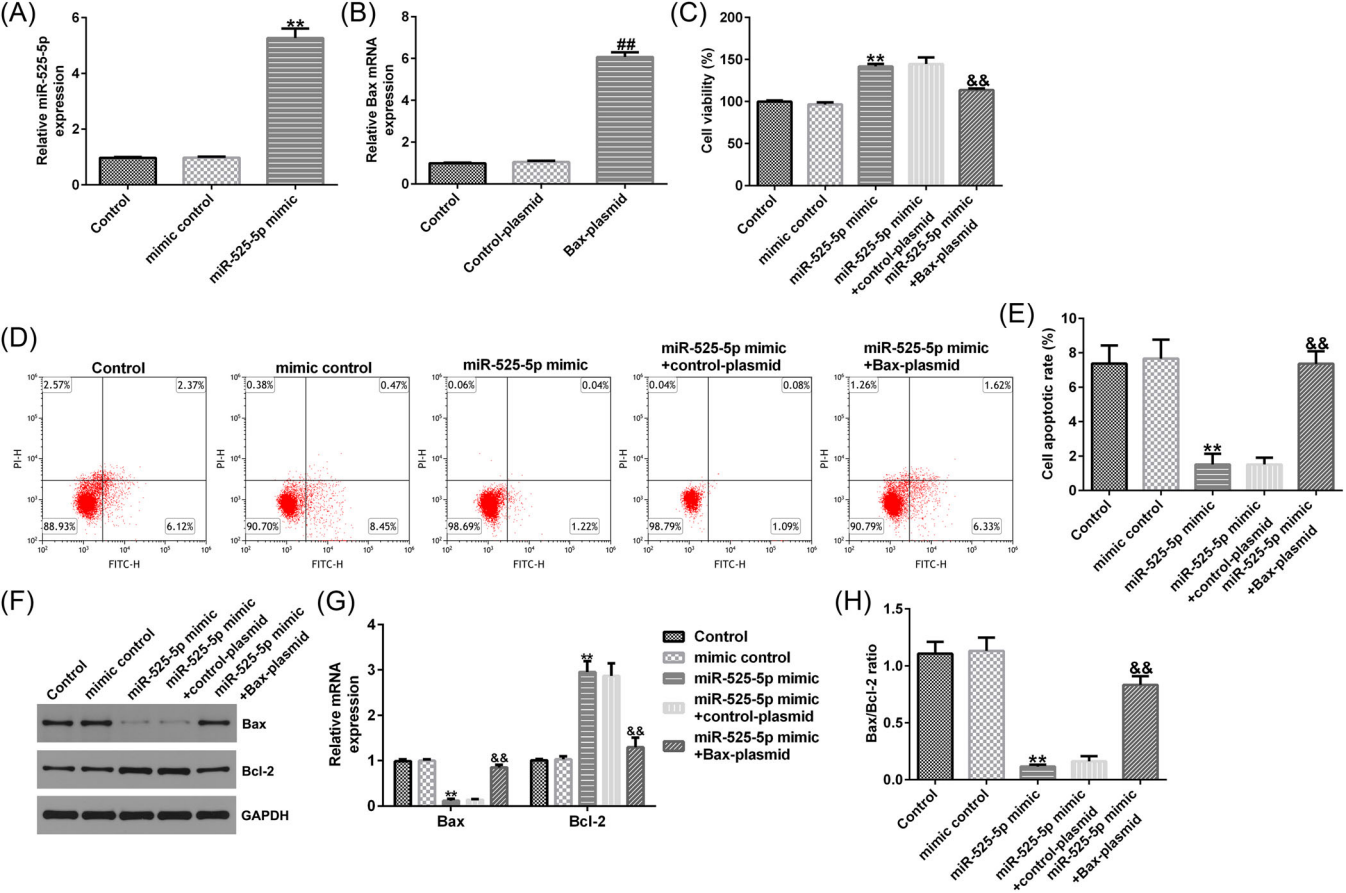
MiR‐525‐5p affects the proliferation and apoptosis of HUVECs by downregulating Bax expression. (A, B) the mRNA levels of miR‐525‐5p and Bax were detected with RT‐qPCR. (C) the proliferation of HUVECs was evaluated with MTT assay; (D, E) flow cytometry was used to detect apoptosis of HUVECs; (F, G). the protein and mRNA levels of Bax and Bcl‐2 were detected by RT‐qPCR and western blot analysis. (H) Bax/Bcl‐2 ratio. **p < .01 versus mimic control; ^##^ *p* < .01 versus control‐plasmid; ^&&^ *p* < .01 versus miR‐525‐5p mimic + control‐plasmid. HUVEC; human umbilical vein endothelial cell; RT‐qPCR, reverse transcriptase‐quantitative polymerase chain reaction.

To further investigate the relationship between miR‐525‐5p and Bax in HUVECs, mimic control, miR‐525‐5p mimic, miR‐525‐5p mimic + control‐plasmid, or miR‐525‐5p mimic + Bax‐plasmid were transfected into HUVECs. The data suggested that miR‐525‐5p mimic notably enhanced the level of miR‐525‐5p in HUVECs, while Bax‐plasmid could increase the expression of Bax in HUVECs (Figure 6A,B). Furthermore, upregulation of miR‐525‐5p in HUVECs remarkably reduced Bax, and Bax‐plasmid could reverse the effect of miR‐525‐5p on Bax (Figure 6F,G). These results demonstrate that miR‐525‐5p negatively regulates Bax expression in HUVECs.

### 3.7 MiR‐525‐5p improves viability and inhibits apoptosis through downregulating Bax expression in HUVECs

We finally detected the functions of miR‐525‐5p on cell proliferation and apoptosis of HUVECs. The findings suggested that miR‐525‐5p mimic significantly enhanced the viability and reduced apoptosis of HUVECs, while Bax‐plasmid significantly abolished this effect (Figure 6C–E). Further analysis of apoptosis‐related genes found that upregulation of miR‐525‐5p could reduce Bax (Figure 6F,G), enhance Bcl‐2 level (Figure 6F,G), and decreased Bax/Bcl‐2 ratio (Figure 6H) in HUVECs. All these effects can be significantly eliminated by Bax‐plasmid (Figure 6C–H). These results indicate that miR‐525‐5p enhances the viability of HUVECs and inhibit apoptosis by downregulating Bax.

## 4 DISCUSSION

DVT is a difficult vascular disease in clinical treatment, and the molecular mechanisms have not been fully elucidated. Currently, the clinical treatment of DVT mainly includes anticoagulants and thrombolytics, but the therapeutic effects are not satisfactory.^1^, ^3^ Studies have found that vascular wall damage is one of the important elements in the formation of DVT, and inhibiting apoptosis and damage of vascular endothelial cells can effectively alleviate DVT.^7^, ^13^ However, the underlying mechanisms need to be further investigated.

Growing evidence suggest that lncRNAs could act as biomarkers for DVT, and are potential therapeutic targets for DVT.^22^ Previous study has identified that LINC00659 is significantly upregulated in patients with DVT through transcriptional profiling, and is an important regulator of DVT development.^22^ However, the roles and regulatory mechanisms of LINC00659 in DVT remained to be further explored. In this study, we found that LINC00659 effectively inhibited the proliferation and promoted the apoptosis of HUVECs. Subsequently, miR‐525‐5p was found to be the direct target of LINC00659 by bioinformatics database, and LINC00659 negatively regulated miR‐525‐5p expression in HUVECs. Downregulation of LINC00659 increased the viability of HUVECs and inhibited apoptosis. However, inhibition of miR‐525‐5p could reverse the roles of LINC00659 downregulation on the proliferation and apoptosis of HUVECs. Further study suggested that miR‐525‐5p directly targeted Bax and negatively regulated Bax expression in HUVECs. In addition, miR‐525‐5p enhanced the viability of HUVECs and inhibit apoptosis by downregulating Bax. These findings elucidate the roles and molecular mechanisms of LINC00659 in DVT.

LINC00659 was found to be involved in varieties of diseases, including cancer and venous thrombosis.^22^, ^23^, ^24^ These studies imply that the mechanism of LINC00659 involved in disease is related to the miRNA regulatory network.^36^ Some research have reported that LINC00659 participates in the occurrence of diseases by regulating cell apoptosis and proliferation. For example, knockdown of LINC00659 could significantly inhibit colon cancer growth by inducing apoptosis.^24^, ^37^ Therefore, we speculated that LINC00659 could regulate the proliferation and apoptosis of venous endothelial cells through targeting miRNAs in DVT.

Research have shown that lncRNAs could act as ceRNAs to sponge miRNAs, thereby regulating the downstream target genes.^38^, ^39^ Our study found miR‐525‐5p is the direct target of LINC00659 in HUVECs, and miR‐525‐5p could negatively regulate Bax expression. MiR‐525‐5p is a miRNA discovered in recent years, which could be involved in the occurrence of various diseases, including myocardial infarction, myocardial hypertrophy, and cerebral ischemia/reperfusion injury.^40^, ^41^, ^42^ MiR‐525‐5p was reported to inhibit cervical cancer metastasis through blocking the UBE2C and ZEB1/2 signaling pathway.^43^ In addition, miR‐525‐5 could regulate epithelial‐mesenchymal transition and cell proliferation in glioma by targeting Stat‐1.^44^ Recently, miR‐525‐5p has been shown to be involved in EPCs angiogenesis in rheumatoid arthritis disease.^45^ Our findings suggest that LINC00659 could participate in the regulation of proliferation and apoptosis of endothelial cells (HUVECs) through regulating miR‐525‐5p/Bax axis. However, it should be mentioned that although HUVECs are venous cell lines, they are not from deep veins (such as human saphenous vein endothelial cells). This was a limitation of current study. Besides, this study was mainly explored at the cellular level, and further studies are needed to enhance the reliability of the results. For example, effect and mechanism of LINC00659 could be verified in vivo by constructing an animal model of DVT. Furthermore, this study did not further validate the relationship between Bax and LINC00659 in DVT. In future studies, we will further explore these issues.

## 5 CONCLUSION

Our study showed that LINC00659 could regulate vascular endothelial cell apoptosis through regulating miR‐525‐5p/Bax axis, thus playing important roles in DVT development. The findings of current study provide new potential target for the treatment and diagnosis of DVT.

## AUTHOR CONTRIBUTIONS

Xizheng Zhu contributed to the study design, data collection, statistical analysis, data interpretation, and manuscript preparation. Beijia Chen contributed to data collection and statistical analysis. Hui Xu contributed to data collection, statistical analysis, and manuscript preparation. All authors read and approved the final manuscript.

## CONFLICT OF INTEREST

The authors declare no conflict of interest.

## Data Availability

Datasets used and/or analyzed during the current study are available from the corresponding author on reasonable request.
